# Ingestion of triglycerides containing medium- and long-chain fatty acids can increase metabolism of ingested long-chain triglycerides in overweight persons

**DOI:** 10.3389/fnut.2023.1260506

**Published:** 2023-11-16

**Authors:** Naohisa Nosaka, Shogo Tsujino, Shohei Sadamitsu, Nanaka Ando, Kazuhiko Kato

**Affiliations:** ^1^Central Research Laboratory, The Nisshin OilliO Group, Ltd., Yokohama, Kanagawa, Japan; ^2^Kato Clinic, Komae, Tokyo, Japan

**Keywords:** medium- and long-chain triglycerides, obesity, metabolism of ingested fat, medium-chain fatty acid, postprandial metabolism

## Abstract

**Introduction:**

Medium-chain fatty acids (MCFAs) have attracted considerable attention for preventing or improving obesity, which is a recognized risk factor for lifestyle-related diseases. Medium- and long-chain triglycerides (MLCTs) are expected to improve the metabolism of ingested long-chain triglycerides (LCTs). However, previous studies have reported mixed results. In this study, the effect of ingestion of MLCTs was evaluated on the metabolism of LCTs and compared to the ingestion of rapeseed oil (control oil).

**Methods:**

A randomized, double-blind, placebo-controlled crossover study was performed among sedentary participants with BMIs ranging from 25 below 30 kg/m^2^. Thirty participants were asked to ingest either 14 g of MLCTs or a control oil for 4 weeks. The metabolism of ingested LCTs was evaluated by measuring isotopically labeled carbon dioxide released by the degradation of carbon-13 (13C)-labeled LCTs.

**Results:**

Ingestion of MLCTs markedly enhanced the metabolism of ingested LCTs by comparison to the control oil.

**Conclusion:**

The findings of this study suggest that ingestion of MLCTs may enhance the metabolism of dietary LCTs through activation of β-oxidation in liver mitochondria, which may increase the metabolic kinetics of ingested long-chain fatty acid (LCFAs).

**Clinical trial registration:**

https://center6.umin.ac.jp/cgi-open-bin/ctr/ctr_view.cgi?recptno=R000053101, identifier: UMIN000046604.

## 1 Introduction

Obesity augments the chances of developing dyslipidemia, hypertension, and type 2 diabetes, which are related to cardiometabolic diseases and metabolic syndrome (1). Mitochondrial dysfunction often occurs in metabolic diseases, including metabolic syndromes (2). Improved mitochondrial function may play an important role in increased energy expenditure and fat oxidation, which contribute to weight loss and maintaining a healthy weight (3). The diet-induced thermogenesis (DIT) and metabolism of ingested LCTs were lower in obese individuals compared to normal weight individuals (4, 5).

MCFAs, comprising unbranched saturated fatty acids with 6–12 carbon atoms, are dietary ingredients that promote improved mitochondrial function (2, 6). As such, ingestion of MCTs, containing 8- and 10-carbon MCFAs, has been investigated. MCTs ingestion is reported to elicit an anti-obesity function by suppressing the accumulation of body and visceral fat (7, 8), enabling postprandial increase in DIT (9–11), and increasing both 24-h energy expenditure (12), fat oxidation during physical activity (13–15), as well as the metabolism of dietary fat (16).

Although MCTs prevent or improve obesity their physicochemical characteristics cause them to smoke during the cooking process at high temperatures (150–200°C). Moreover, heating a mixture of cooking oil and MCTs together causes foaming. To resolve these issues, we prepared MLCTs by mixing 80%–90% rapeseed oil and 10%–20% MCTs before initiating an enzymatic catalyzed ester exchange reaction. The net result of this reaction was to randomize the fatty acids on the glycerides. These MLCTs increase the smoke point and reduce foaming during cooking. Moreover, these improvements allow MLCTs to be regularly used in the same wide range of cooking applications as fats and oils (17). Human studies have been conducted to analyze the ingestion of MLCTs. Akin to the results for MCTs, MLCTs ingestion was found to inhibit the accumulation of body fat and visceral fat (18–21), enhance postprandial DIT (22, 23), and increase the oxidation of fat during physical activity (24). Results from animal studies suggest ingestion of MLCTs increase the hepatic degradation of LCFAs (25, 26). Therefore, MLCTs are expected to act in the same way as MCTs to improve the metabolism of ingested LCTs. However, previous studies using butter and coconut oil (which contain small amounts of octanoic and decanoic acids) have reported mixed results (27, 28).

Based on these results, the present study was designed to establish whether 4-week ingestion of MLCTs in humans enhances the metabolism of ingested fat. Specifically, overweight volunteers were selected. Obesity is defined as a BMI of 25 kg/m^2^ or more (29) and BMIs ranging from 25 below 30 kg/m^2^ is classified as obese class-1 (30) in Japan. According to this definition, about 30% of men and 20% of women in Japan are considered obese (31). After the initial 4 week feeding period, metabolism of dietary fat was investigated using isotopically labeled LCTs by monitoring the release of labeled carbon dioxide.

## 2 Methods

### 2.1 Ethical considerations and participant

This study complied with the Declaration of Helsinki. The investigation was performed with assistance from a physician at the Kouwa Clinic, Kouwa-kai Medical Corporation (Tokyo, Japan) and Fuji Medical Science Co., Ltd. (Chiba, Japan) as a contract research organization (CRO, Huma R&D Co., Ltd., Tokyo, Japan). The proposed experimental procedures were reviewed by the Yoga Allergy Clinic Clinical Research Ethics Review Committee (approval number: RD10012KW04). This study was registered with UMIN-CTR prior to initiating the investigation (UMIN CTR ID: UMIN000046604).

Thirty volunteers were recruited for this study. Previous studies have examined the fat oxidation rate during physical activity (15) and metabolic rate of dietary fat after meals (16) containing 2 g of MCTs for 2 weeks in overweight persons. The least number of subjects required for this analysis was evaluated to be 28.

Volunteers were recruited from the participant registration bank of the contract research organization. Potential subjects were screened by conducting an interview, testing blood pressure, and performing a range of biochemical analyses of the blood. Participants were eligible for the study if: (i) selection criteria were met, (ii) administrative requirements throughout the study period could be fulfilled, and (iii) involvement in the study was judged by the principal investigator to be safe.

A double-blind study was performed using a crossover method. Participants were randomly allocated into two groups. One group was given MLCT first (MLCT-first group) and the other group received the control food first (control-first group). The two groups were equivalent with respect to sex and age. The washout period was 5 weeks between the two intervention periods (period-1 and period-2). The schedule for this study is shown below.

**Figure:**
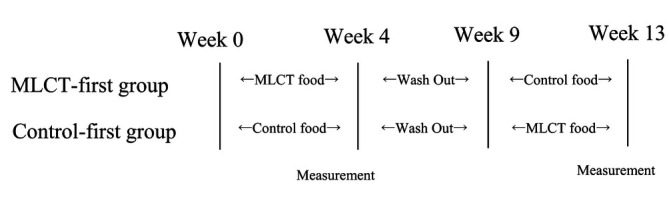


### 2.2 Test food

MLCTs (Nisshin OilliO Group, Ltd., Tokyo, Japan) was used as the test oil, and rapeseed oil (Nisshin OilliO Group, Ltd.) was used as the control oil. The fatty acid composition of their oils is shown in Table 1. The MLCTs comprised inter-esterified triglycerides containing 1.6 g of MCFAs in 14 g (32). During the intervention period, test foods containing MLCTs (MLCT food), or control oil (control food) were ingested (303 kcal energy, 8.5 g protein, 14.3 g fat, 35.1 g carbohydrate). Participants who ingested MLCT food comprised the MLCT group, and those who ingested control food comprised the CO group. Both the test and control food diets were ingested for 4 weeks. Throughout the study period patients were asked to continue their usual lifestyle and not to engage in vigorous exercise, eating small meals, overeating, and excessive consumption of alcohol.

**Table 1:** The fatty acid composition of the control and test oils.

g/100 g fatty acids	Control oil	Test oil
Saturated fatty acids	9.0	19.5
Medium-chain fatty acids	0.0	11.6
Mono-unsaturated fatty acids	63.9	56.5
Poly-unsaturated fatty acids	27.2	24.0
n-6 fatty acids	19.5	17.2
n-3 fatty acids	7.7	6.8

### 2.3 Nutritional survey

On the 25th, 26th, and 27th days following ingestion of the MLCT or control food, the participants were asked to photograph their meals and record details on a survey form over the three day period. From the information provided by the participants, nutrient calculations were performed to determine the daily intake of energy, protein, fat, carbohydrate, saturated fatty acids, MCFAs, monounsaturated fatty acids, polyunsaturated fatty acids, n-6 polyunsaturated fatty acids, and n-3 polyunsaturated fatty acids (33).

### 2.4 Measurement

After ingesting the test foods for 4 weeks and then fasting overnight, metabolic rate was measured in the laboratory. Here, participants were asked to enter relevant details in the human calorimeter. The accurateness of the readings were confirmed as previously reported (15, 16). During the study period both oxygen consumption and carbon dioxide production recoveries were within 100 ± 2%.

Each participant ingested a test meal containing 14 g of either MLCTs or the control oil and 400 mg of 13C-labeled triolein (481 kcal energy, 22.4 g protein, 18.7 g fat, 57.6 g carbohydrate). 13C-labeled triolein was TRIOLEIN (1,1,1–13C3; Cambridge Isotope Laboratories, Inc., Tewksbury, MA, United States).

Participants rested for 4 h before and after ingestion of the test meal. Exhaled breaths were collected with a gas bag approximately every hour before and after ingestion of the test meal.

### 2.5 Experimental determination of the metabolic rate of ingested LCTs

Metabolism of dietary fat was determined from the rate of excretion of 13C-labeled carbon dioxide. The metabolic rate of ingested 13C-labeled triolein was determined using the same formula as previously reported, and cumulative values were calculated using the same method (15).

### 2.6 Statistical analysis

Data for participant period-1 or period-2 were analyzed. When missing values occurred, multiple imputations were conducted. Dietary intake and body weight during the intervention period were checked for normality by the Shapiro–Wilk test to evaluate the two intervention groups (MLCT and CO groups). Cumulative metabolic rate of ingested LCTs at 4 h were postprandial. For cases of no normality, the Mann–Whitney U test was performed, and if normality was found, equal variances were confirmed by an F test. Student’s *t*-test was performed if there was equal variance, and Welch’s t-test was used if there was no equal variance.

The intervention effect values for the cumulative metabolic rate of ingested LCTs at 4 h postprandial (values for the MLCT group minus values for the CO group) were confirmed for normality by the Shapiro–Wilk test. If no normality was found, a Mann–Whitney U test was performed. If there was normality, a linear mixed model was used. Restricted maximum likelihood estimation was performed with a random intercept model equation with group, time, and order of intake as fixed effects and participants as variable effects. Significance, estimates, and 95% confidence intervals were obtained for fixed effects. Measurement indicators that showed significant differences were analyzed for carryover effects, and results were withheld if the significance of the difference was found.

Basic statistical analysis was performed using Microsoft Excel for Office365 (Microsoft Japan Co., Ltd., Tokyo, Japan). All statistical processing was done using the R package (ver. 4.1.0; R Core Team, Vienna, Austria). Note, *p*-values of <5% indicated a significant difference.

## 3 Results

### 3.1 Participant

In all, 80 individuals who gave informed consent were evaluated for eligibility to participate in the study. Of these, 33 were excluded (4 for not meeting the selection criteria, 4 due to their own free will, 8 for personal reasons other than their own free will, and 17 for other reasons). The remaining 47 eligible participants were randomly split into two groups: 24 in the MLCT-first group and 23 in the control-first group.

After commencement of the study, 1 participant in the MLCT-first group discontinued before the period-1 visit and 1 participant in the control-first group dropped out of the study. In all, 15 participants from each group were analyzed in period-1 (i.e., the target number of patients, 30, was measured). A total of 14 participants, 7 participants in each group, were not measured at the end of period-1. One participant in the MLCT-first group discontinued before coming to the hospital for period-2, and 14 participants in the MLCT-first group and 15 participants in the control-first group were evaluated in period-2. Thus, measurement data was obtained from 30 participants in the MLCT group and 29 participants in the CO group. All reasons for discontinuation were related to restrictions on outdoor activity due to the COVID-19 pandemic. Subject details are shown in Table 2.

**Table 2:** Details of the participants (male *n* = 14, female *n* = 16).

	*n* = 30		
Age, year	50.5	±	8.0
Height, cm	164.7	±	6.1
Weight, kg	73.6	±	7.0
BMI, kg/m^2^	27.0	±	1.4

Values are expressed as mean ± SD.

No adverse events attributable to the ingestion of the test food (including the MLCT food and the control food) were observed during the study period. Two participants in the CO group each reported one adverse event (1 with diarrhea; 1 with bloating, belching, and soft stools) that could have been caused by the ingestion of the control food. However, the physician decided to continue the study because these adverse events are commonly observed following the ingestion of foods.

There were 30 participants in the analysis with measurement data from either period-1 or period-2. Data analysis was performed after multiple imputations to the nutrient intake and cumulative metabolic rate data of dietary LCTs for 1 participant who discontinued the study in period-2.

### 3.2 Nutrient intake

Nutrient intakes based on dietary surveys during the intervention period are shown in Table 3, the calculated nutrient intakes include the test foods. A difference in intake of MCFAs was seen between the two groups, reflecting the intake of the different test foods.

**Table 3:** Nutrient intake during the intervention period.

	CO group			MLCT group		
Energy, kcal	2035	±	58	2029	±	62
Protein, g	70.3	±	2.7	71.8	±	2.4
Fat, g	80.5	±	3.6	80.6	±	3.4
Carbohydrate, g	247	±	8	244	±	9
Saturated fatty acids, g	19.6	±	1.1	20.7	±	1.1
Medium-chain fatty acids, mg	276	±	36	1925	±	53^*^
Mono-unsaturated fatty acids, g	31.3	±	1.5	31.5	±	1.3
Poly-unsaturated fatty acids, g	20.0	±	0.8	18.8	±	0.8
n-6 fatty acids, g	16.3	±	0.7	14.9	±	0.7
n-3 fatty acids, g	3.5	±	0.2	3.7	±	0.2

Values given as mean ± SD. The calculated nutrient intakes include the test foods. * Significant difference from CO group (*p* < 0.05).

### 3.3 Metabolic rate of ingested LCTs

TRIOLEIN (1,1,1–13C3) was ingested at 2.14% (w/w) per fat in meal, at an average of 5.4 mg/kg body weight per participant. The aggregate metabolic rate of dietary LCTs for each diet group and their intervention effect values are shown in Table 4. The intervention effect value for the cumulative metabolic rate of dietary LCTs was significantly higher in the MLCT group than in the CO group (*p* < 0.05, Mann–Whitney’s U test, Table 4).

**Table 4:** Cumulative value of metabolic rate of dietary LCTs.

	Cumulative value (4 h of AUC)							Intervention effect			
	CO group				MLCT group			(MLCT-CO)			
Metabolic rate of ingested LCTs, %	1.8	±	0.3		2.3	±	0.3	0.5	±	0.3	#

Values given as mean ± SE. # Significant difference (*p* < 0.05, Mann-Whitney’s U test).

## 4 Discussion

When verifying the effectiveness of dietary elements in combating obesity, major techniques for analyzing changes in metabolism after a meal include energy expenditure (which may be distinguished by fat and carbohydrate oxidation), the dynamics of blood lipids, and the degradation of specific fatty acids using 13C isotope labeling. Numerous studies indicate augmented postprandial energy expenditure utilizing MCTs and MLCTs (9–12, 22, 23, 34). Conversely, there are conflicting conclusions concerning fat and carbohydrate oxidation reported across studies. MCTs effectively reduce postprandial chylomicron concentrations and hinder the elevation of TG concentrations in blood lipids (35–37). In contrast, no noticeable differences result on the blood lipid from the ingestion of MLCTs [coconut oil (38)], as they contain LCFAs within the MLCTs. To differentiate fat metabolism in the body from the metabolic process of ingested fat, the optimal method is considered to evaluate the decomposition of targeted fatty acids via ingested 13C isotopes. Studies analysing the degradation of 13C-labelled fatty acids have demonstrated a distinct escalation in the oxidation of MCFAs (5, 39). However, a growth in the metabolism of ingested LCTs has only recently been documented when ingested MCTs (14) but not MLCTs. Previous studies of short-term (11 days) butter ingestion have shown increased net postprandial oxidation of dietary myristic acid but not palmitic acid, depending on the fatty acid composition of the test meal, compared with tallow ingestion (27). The combined ingestion of butter and coconut oil for 14 days increased postprandial net saturated LCFAs oxidation but did not increase dietary saturated LCFAs oxidation more than tallow ingestion (28).

Here, we evaluated metabolism of ingested 13C-labeled triolein in overweight participants following a 4-week period of ingestion of either MLCTs or control oil. The findings indicated that participants in the MLCT group displayed significantly increased metabolism of ingested triolein compared to those given control oil (CO group). This study found similar results for increased metabolism of ingested LCTs to those reported from studies with small amounts of MCTs ingested (16), but different results from those with butter and coconut oil ingested (27, 28). The fat energy in the test meals in which postprandial metabolism was assessed in the studies with MCTs and MLCTs (this study) was 7%–8% per day, whereas the test meals in the studies with butter and coconut oil were high in fat energy, around 30%–50%. It is speculated that differences in the amount of total fat in the test meals may have affected the metabolism of ingested fat after the meal. In addition, the present study investigated the effect of fats and oils high in unsaturated fatty acids, comparing rapeseed oil and its inter-esterified counterpart, MLCTs. While previous studies using butter and coconut oil have examined the metabolism of ingested saturated LCFAs in the diet of mainly saturated fats and oils, this study measured the metabolism of oleic acid as a monounsaturated fatty acid in the diet. It has been discussed that the degree of postprandial oxidation varies with the type of fatty acid, and it is generally thought that the increase in postprandial oxidation is saturated fatty acids < monounsaturated fatty acids < polyunsaturated fatty acids. However, the differences between these fatty acids are not clear, as some studies have found no difference between saturated and monounsaturated fatty acids (40).

The present investigation was conducted using a crossover study design, with no significant differences in energy and energy-producing nutrient intake between the two groups. Furthermore, the analysis of the carryover effect with respect to the influence of the order of allocation was not significant. These findings confirmed that there was no significant difference between the effects of energy and nutrient intake and allocation during the test food ingestion period, and that the results obtained were due to the effects of MLCTs ingestion. In this study, we used rapeseed oil as the control oil. Rapeseed oil is the most widely used edible oil in Japan. Therefore, the test oil, which was prepared by inter-esterification of rapeseed oil and MCTs, was considered to be highly acceptable to the Japanese and easy to substitute. As shown in the Table 1, rapeseed oil contains n-3 and n-6 polyunsaturated fatty acids in addition to monounsaturated fatty acids. MLCTs reduce the monounsaturated fatty acids and n-3 and n-6 polyunsaturated fatty acids that are provided by consuming rapeseed oil, depending on the amount of MCFAs. However, rapeseed oil and MLCTs have generally equivalent cooking oil properties. Replacing edible oil with MLCTs, which do not differ markedly except for containing the low-level of MCFA, would increase the metabolism of ingested LCTs, as shown in this study, suggesting that ingestion of MLCTs may help prevent or ameliorate obesity.

Previous studies allow for inference that the fatty acid degrading system is activated in the liver due to the functional properties of the MLCTs obtained in this study. Ingested LCTs are absorbed from the intestinal tract as LCFAs and LCFA-bound monoacylglycerol, which are then resynthesized into LCTs to form lipoproteins and released into the blood via the lymphatic system. In the bloodstream, LCTs are degraded by lipoprotein lipase and converted to LCFAs, which are then transferred to adipose tissue, muscle tissue, and the liver (i.e., major organs in the body that utilize LCFAs) (40, 41). Crucially, the liver actively expends energy, even at rest after eating (42), and dietary LCFAs are oxidized when they reach the liver. Previous animal studies examined the effects of MLCTs ingestion on hepatic β-oxidation activity (25). Specifically, rats were fed either MLCTs or LCTs over a 4 week period. The expression of long-chain acyl-CoA dehydrogenase at the mRNA level in the MLCTs group was significantly higher compared to LCTs group, while PPARα mRNA expression was the same in both groups. This observation suggested that the increase in peroxisomal fatty acid oxidation may be unaffected by the ingestion of LCTs. In another study involving rats, the activity of liver acyl-CoA dehydrogenase was measured 1 h after a single ingestion of either MLCTs or LCTs and palmitoyl-CoA as substrate (26). Long-chain acyl-CoA dehydrogenase enzyme activity was significantly elevated together with the corresponding level of mRNA in the MLCTs group compared to the LCTs group. Taken together, these results suggest that the activity and corresponding gene expression of enzymes that oxidize LCFAs in the mitochondrial β-oxidation system may be increased in the liver of animals fed MLCTs, and that the oxidation of LCFAs is accelerated in the liver. MCFAs absorbed from the intestinal tract are not as easily resynthesized into triglycerides as LCFAs, and most of MCFAs are quickly metabolized in the liver (6). Studies in animals have also shown that the oxidation of MCFAs (i.e., octanoic acid) in hepatocytes is five-fold faster than that of LCFAs (i.e., oleic acid), partly because MCFAs and albumin exist in a bound state at a lower ratio than LCFAs and are more readily taken up by the hepatocytes (43). Indeed, previous human studies have shown that ingested MCTs are oxidized at a faster rate than ingested LCTs (5, 39). Furthermore, previous animal studies have shown mRNA expression of long-chain acyl-CoA dehydrogenase was significantly upregulated after ingestion of both MCTs and MLCTs instead of LCTs (26). The post-absorption metabolic kinetics of MCFAs may also alter the activation of fatty acid oxidation of LCFAs in the liver.

A limitation of this study was that it exclusively involved Japanese participants. Thus, it is unclear whether similar results would be obtained for other ethnicities. Moreover, Asians reportedly differ from Caucasians in body composition, as evidenced from the difference in body fat percentage and fat-free mass (44). Consequently, it is not obvious whether similar results would be obtained if different dietary compositions were used to assess metabolism of ingested fat. Another limitation of the study is that it did not examine populations with different exercise habits or higher levels of obesity.

## 5 Conclusion

The present study showed that 4 weeks of MLCTs ingestion could significantly enhance the metabolism of ingested LCTs in overweight individuals with no exercise habits. Our findings suggest that MCFAs exhibit different metabolic kinetics than LCFAs and contribute to enhanced degradation of ingested fatty acid by activating the β-oxidation system in liver mitochondria.

At present, people who need fats and oils with metabolic functions select and consume them, but in the future, edible oils will be developed that contain fatty acids with metabolic functions that are needed by each individual, such as MLCTs. With progress, it may be possible to consume optimal fats and oils without increasing fat intake.

## Data availability statement

The datasets presented in this article are not readily available because data not available due to commercial restrictions. Requests to access the datasets should be directed to NN, n-nosaka@nisshin-oillio.com.

## Ethics statement

The studies involving humans were approved by Yoga Allergy Clinic Clinical Research Ethics Review Committee. The studies were conducted in accordance with the local legislation and institutional requirements. The participants provided their written informed consent to participate in this study.

## Author contributions

NN: Conceptualization, Methodology, Writing – original draft. ST: Conceptualization, Writing – review & editing. SS: Formal analysis, Writing – review & editing. NA: Formal analysis, Writing – review & editing. KK: Supervision, Writing – review & editing.
